# The Plight of the Scottish Jubilee Institute

**Published:** 1921-01-01

**Authors:** 


### The Plight of the Scottish Jubilee Institute.

The Scottish branch of Queen Victoria's Jubilee Institute meeting at Glasgow recently were called on to consider a really disastrous financial situation. The deficit on the year's work amounts to over £6,000. The value of the investments has seriously depreciated, and, when the bank has been repaid, will stand at £8,800 out of a present face value of £19,000. There is barely sufficient capital to carry on the current year. At a conference of district nursing associations held prior to the annual meeting, the Countess of Eglinton and Winton presided, and it was stated that a general public appeal for funds would be launched in January. Unless it succeeds, Mr. John S. Pitman declared they would be "on the rocks." Sir Donald MacAlister, who presided at the annual meeting, gave an account of the work carried on in the 313 nursing associations affiliated with the Scottish branch. The number of applications from fully-trained nurses for district and midwifery training has largely increased. The demand for nurses is so great that the Institute now trains eighty-three, as compared with fifty before the war. The supply is largely drawn from those recently demobilised, and Sir Donald stated they had the confidence and sympathetic support of the whole medical profession and of the public in Scotland. He urged that the Institute furnished a suitable basis for a national nursing system. Under such a system, which would consolidate and unify the work of district nursing and health visiting, they could reduce the number of officials required and the system under which the same families were visited for different purposes day after day. Dr. Madeline Archibald, who declared from her own experience that industrial practice would be a nightmare if it were not for the Jubilee nurses, urged that the Ministry of Health should invite a committee of panel practitioners and district nurses to go to the crux of the whole problem; no scheme could succeed which was not based on close cooperation between the practitioners and the district nurses.

The proposal to make a charge for the nurses' services found no very warm supporters. If it be true that the fundamental purpose of the Institute is to secure that the necessitous poor shall be nursed free of charge, it ought to be recognised that social conditions have changed since the days of Queen Victoria.

